# Effectiveness and safety analysis of Danggui Shaoyao Powder for the treatment of non-alcoholic fatty liver disease: study protocol for a randomized, double-blind, placebo-controlled clinical trial

**DOI:** 10.1186/s12906-023-03948-3

**Published:** 2023-04-19

**Authors:** Qian Huang, Ziming An, Xin Xin, Qinmei Sun, Siting Gao, Sheng Lv, Xiao Xu, Shuohui Yang, Fang Lu, Jie Yuan, Yu Zhao, Yiyang Hu, Ping Liu, Qin Feng

**Affiliations:** 1 Institute of Liver DiseasesShuguang Hospital Affiliated to Shanghai University of Traditional Chinese Medicine, Shanghai, China; 2 Shanghai Key Laboratory of Traditional Chinese Clinical Medicine, Shanghai, China; 3 Key Laboratory of Liver and Kidney Diseases, Shanghai University of Traditional Chinese Medicine, Ministry of Education, Shanghai, China; 4 Department of Radiology, Shanghai Municipal Hospital of Traditional Chinese MedicineShanghai University of Traditional Chinese Medicine, Shanghai, China; 5 Department of Radiology, Shuguang Hospital Affiliated to Shanghai University of Traditional Chinese Medicine, Shanghai, China

**Keywords:** Danggui Shaoyao Powder, Non-alcoholic fatty liver disease, Randomized controlled trial, Traditional Chinese medicine

## Abstract

**Background:**

The incidence of non-alcoholic fatty liver disease (NAFLD) has been on the rise in recent years, and there are no effective drugs to treat NAFLD; therefore, effective prevention and treatment of NAFLD have become a new challenge. Danggui Shaoyao Powder (DGSY) is a classic prescription commonly used in clinical practice and has been shown to reduce hepatic steatosis in patients with NAFLD. In addition, previous studies have shown that DGSY can alleviate hepatic steatosis and inflammation in NAFLD mice. Although clinical practice and basic studies have shown that DGSY is effective in NAFLD, high levels of clinical evidence are lacking. Therefore, a standardized RCT study protocol is required to evaluate its clinical efficacy and safety.

**Methods and analysis:**

This study will be a randomized, double-blind, placebo-controlled, and single-center trial. According to the random number table, NAFLD participants will be randomly divided into the DGSY or placebo group for 24 weeks. The follow-up period will be 6 weeks after drug withdrawal. The primary outcome is the relative change in MRI-proton density fat fraction (MRI-PDFF) from baseline to 24 weeks. Absolute changes in serum alanine aminotransferase (ALT), liver stiffness measurement (LSM), body mass index (BMI), blood lipid, blood glucose, and insulin resistance index will be selected as secondary outcomes to comprehensively evaluate the clinical efficacy of DGSY in the treatment of NAFLD. The safety of DGSY will be evaluated by renal function, routine blood and urine tests, and electrocardiogram.

**Discussion:**

This study will provide evidence-based medical corroboration for the clinical application of DGSY and promote the development and application of this classic prescription.

**Trial registration:**

http://www.chictr.org.cn. Trial number: ChiCTR2000029144. Registered on 15 Jan 2020.

**Supplementary Information:**

The online version contains supplementary material available at 10.1186/s12906-023-03948-3.

## Introduction

Liver steatosis > 5% is defined as Non-alcoholic fatty liver disease (NAFLD) [1]. The disease spectrum includes non-alcoholic fatty liver (NAFL), non-alcoholic steatohepatitis (NASH), and related liver fibrosis and cirrhosis [2, 3]. NAFLD can not only cause end-stage liver disease and death but is also closely associated with a higher incidence of metabolic syndrome (MetS), type 2 diabetes mellitus (T2DM), arteriosclerotic cardiovascular disease, and colorectal tumors [4]. According to epidemiological statistics and analysis, the prevalence rate of NAFLD among adults in the world is as high as 25%, and that in Western countries is about 17%-46% NASH has become the leading cause of hepatocellular carcinoma in the United States and most common reason for liver transplantation [5, 6]. Therefore, active prevention and control of NAFLD are of great social significance.

The etiology of NAFLD is complex, and its exact pathogenesis has not been fully elucidated. Currently, no drug has been approved by the Food and Drug Administration to treat NAFLD. “Control your mouth, take your legs” is an important therapeutic measure to prevent and treat NAFLD, but patients often have difficulty controlling their diet and maintaining their ideal weight. In addition, obeticholic acid, which has completed Phase III trials as a farnesoid X receptor (FXR) agonist that improves insulin sensitivity and reduces hepatic steatosis, inflammation, and fibrosis, showed promising results in NASH; however, pruritus was present in 23% of treated patients, and low-density lipoprotein cholesterol (LDL-C) was rapidly elevated in some patients. Therefore, drug safety requires long-term evaluation [7]. Given the high incidence of NAFLD and the lack of specific therapeutic drugs, the research and development of new drugs for NAFLD is particularly important.

Traditional Chinese medicine (TCM) has been widely used in China. As a drug treatment approach for NAFLD, TCM is effective in promoting the reversal of NAFLD and improving clinical symptoms and laboratory indicators and is likely to provide a comprehensive therapeutic advantage in the treatment of NAFLD. Danggui Shaoyao Powder (DGSY, composed of Danggui, Shaoyao, Fuling, Baizhu, Zexie, and Chuanxiong) was obtained from the *Synopsis Golden Chamber.* Previous study by other researchs found that DGSY could decrease ALT and improve hepatic fat deposition assessed by elastography in NAFLD patients [8]. Animal studies also revealed that it could alleviate hepatic fat deposition and inflammation in NAFLD mice [9]. However, the current relevant clinical trial evidence levels are insufficient.

Therefore, this study will systematically evaluate the clinical efficacy and safety of DGSY in treating NAFLD through a prospective design and a randomized, double-blind, placebo-controlled, single-center trial.

## Methods

### Study design overview

**Fig. 1:**
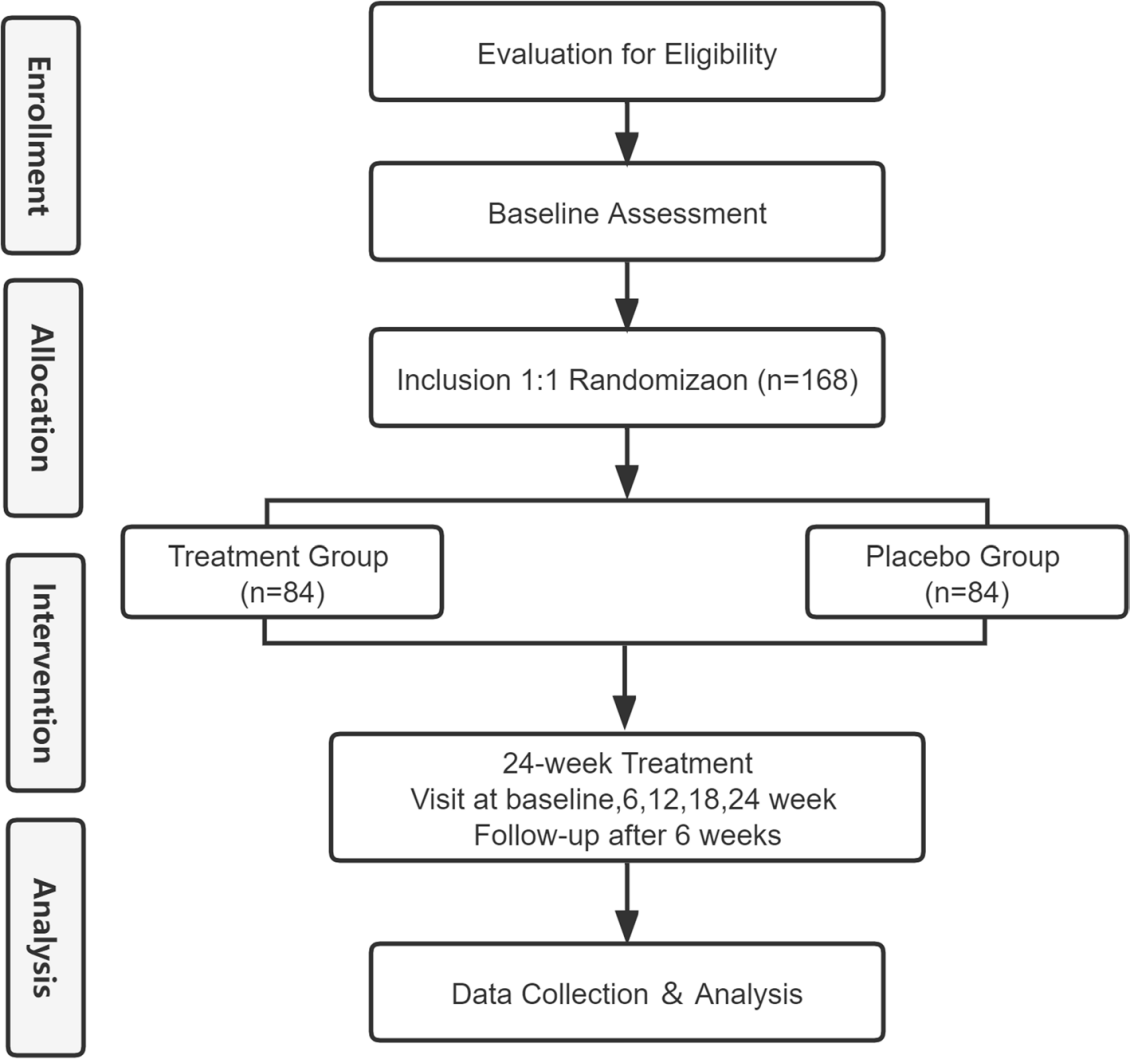
Flow diagram of the study

This is a randomized, double-blind, placebo-controlled, and single-center trial protocol. This clinical trial was registered at Chictr.org.cn (ChiCTR2000029144). A brief flowchart of the study flow is presented in Fig. 1. The Standard Protocol Items: Recommendations for Interventional Trials (SPIRIT) [10] Checklist is shown in Additional file 1. Our study will be conducted at Shuguang Hospital, affiliated with the Shanghai University of Traditional Chinese Medicine. Totally, 168 patients will be randomly assigned to receive DGSY or a placebo for 24 weeks. Clinical efficacy and safety measures will be collected at baseline, weeks 6, 12, 18, and 24, and follow-up.

### Subjects

#### Recruitment

Eligible participants will receive basic information about the study through recruitment advertisements or web messages posted by the researchers and then contact the investigators. Participants will be informed of the trial’s possible benefits and potential risks and will be presented with informed consent to ensure that participation is completely voluntary. After signing the informed consent form, the participants will be successfully recruited. Disease-related biomarkers, imaging examinations, and quality-of-life questionnaires were administered during enrollment. The results will be recorded confidentially in the Case Report Form (CRF).

#### Diagnostic criteria

The diagnostic criteria for NAFLD refer to “The Guidelines for the Prevention and Treatment of Non-alcoholic Fatty Liver Disease (2018, China)” [11]. NAFLD can be diagnosed by imaging or histopathology. Imaging diagnoses included B-ultrasound, MRI-PDFF (> 5%), and FibroScan (controlled attenuation parameter (CAP) > 240 db/m). It can also be diagnosed by histopathology with > 5% hepatocyte steatosis. However, specific diseases that can cause fatty liver were excluded.

#### Inclusion criteria

(1) Meet the diagnostic criteria for NAFLD; (2) Age between 18 and 65 years; (3) MRI-PDFF ≥ 10%; (4)1ULN ≤ ALT ≤ 5ULN; (5) Signed patient informed consent.

#### Exclusion criteria

(1) hepatoprotective and enzyme-lowering drugs in the past 3 months that affect the efficacy evaluation; (2) alcoholic fatty liver, cirrhosis, hepatitis B/C, autoimmune liver disease, or drugs that may cause fatty liver disease; (3) normal liver function; (4) patients who have undergone gastrointestinal bariatric surgery in the last year or who have taken weight-loss drugs in the last 3 months have lost > 10% of their body weight; (5) pregnant women, lactating women, and those with cardiovascular, lung, kidney, hematopoietic system, other primary diseases, malignant tumors, and other serious diseases.

#### Withdrawal criteria and termination criteria

Under patient management protection rules, patients have the right to withdraw from the study for any reason. For patients who propose to withdraw from the experiment midway, the reasons should be clearly recorded in detail as the evaluation index at the time of termination. If a patient fails to return to the hospital on time, researchers should try to inquire about the reason by telephone or letter to investigate the curative effect of the medication. Physicians participating in clinical trials should carefully record the reasons for the termination of the trial and the relationship with the trial and analyze the possible impact of the terminated cases on the research conclusion. The original data of all eliminated and terminated cases should be kept and filed for review after the end of the trial. In addition, treatment was discontinued in patients with the following events: (1) any anticipated adverse event; (2) hepatic decompensation occurring during the enrollment period; (3) compliance < 80% or > 120%, disobedience to treatment; (4) pregnancy in this study; (5) adverse events that are intolerable (patients or investigators may decide to withdraw from the study); (6) diseases or factors unrelated to treatment; (7) loss of follow-up; and (8) breaking blind cases.

### Sample size

The sample content required by each experimental group is calculated according to the sample content estimation method of randomized controlled trials. The calculation formula is as follows:

$${N}_{T}={N}_{C}=\frac{{\left({u}_{\alpha /2}+{u}_{\beta }\right)}^{2}}{2\times {\left(\mathrm{arcsin}\sqrt{{P}_{2}}-\mathrm{arcsin}\sqrt{{P}_{1}}\right)}^{2}}$$

According to the literature [12], the effective rate of the main efficacy evaluation index MRI-PDFF in the control group (routine lifestyle intervention) is 12%, and the effective rate in the DGSY group is increased to approximately 30.3% according to the preliminary clinical observation expectation. Assuming α = 0.05, β = 0.20, P_1_ = 0.303, P_2_ = 0.12, *N*_*T*_: *N*_*C*_ = 1. The sample size of each group is 75 cases, and it was estimated that there would be 84 cases in each group based on the 10% shedding rate. A total of 168 patients with non-alcoholic fatty liver disease were enrolled in the study.

### Randomization, allocation, and blinding

The central randomized system of Data Acquisition System (DAS) clinical trials will be used to apply for random numbers and dispense drugs. Patients will be randomly divided into treatment and control groups in a ratio of 1:1. All participants and investigators will be blinded to the treatment assignment until the end of the study period. The first and second blind bases, made according to a random number, shall be duplicated and properly kept. The researcher shall not damage or open the paper at will. Unblinding was performed twice. The first de-blinding process decomposed the assigned groups (Groups A/B) without the specified intervention name. Statisticians will perform statistical analyses without knowledge of the interventions. After analysis, a second unblinding was performed to determine which group (Group A/B) was DGSY/placebo. All unblinding processes were recorded. In an emergency, the reason, time, and place of blindness will be recorded and treated as blindness withdrawal.

### Intervention

Lifestyle intervention: The enrolled patients will be treated by repeated health education, diet abstinence, increased exercise, modification of bad behavior, and other basic lifestyle treatments. (1) Low sugar, low fat, and high vitamin diet will be recommended; (2) Strengthening physical exercise is recommended; (3) Adjusting emotions and keeping the mood comfortable; and (4) Other traditional Chinese medicine and Western medicine, mainly used to treat fatty liver or lipid-lowering will be prohibited during the trial period.

The participants assigned to intervention group will take DGSY granules (8.4 g/pack × 2 packs, bid) and those assigned to placebo group will take placebo granules (2 packs, bid) for 24 weeks. According to the initial prescription dose and clinical treatment practice of NAFLD, the daily raw drug dose for adults in the DGSY group was *Angelica sinensis.* (Danggui) 9 g, *Angelica sinensis.* (Shaoyao) 20 g, *Wolfiporia cocos.* (Fuling) 12 g, *Atractylodes macrocephala.* (Baizhu) 12 g, *Alismatis Rhizoma.* (Zexie) 20 g; and *Ligusticum wallichii Franch.* (Chuanxiong) 12 g. The DGSY composition is shown in Table 1.

**Table 1:** The composition of DGSY

Herb	Latin scientific name	Dosage(g)	Produced from
Dang Gui	*Angelica sinensis*	9	Dried root
Shao Yao	*Paeonia lactiflora Pall*	20	Dried root
Fu Ling	*Wolfiporia cocos*	12	Dried sclerotium
Bai Zhu	*Atractylodes macrocephala*	12	Dried rhizome
Ze Xie	*Alismatis Rhizoma*	20	Dried tuber
Chuan Xiong	*Ligusticum wallichii Franch*	12	Dried rhizome

Placebo treatment: placebo granules contained 5% DGSY granules and 95% other ingredients (contains 98.77% maltodextrin, 0.75% caramel pigment, 0.15% lemon yellow pigment, 0.03% sunset yellow pigment, and 0.30% bitters). The pellets were masked with the same packaging and a similar taste as DGSY. Both DGSY and placebo will be manufactured and packaged by Tianjiang Pharmaceutical Co. Ltd., Jiangyin City, Jiangsu Province, China.

### Study visit overview

Participants will be followed-up before enrollment and 6, 12, 18, 24, and 30 weeks after enrollment. Each follow-up date will have a time flexibility of 5 days. Vital signs were recorded at each follow-up. MRI-PDFF will be performed at weeks 0 and 24, and the blood indicators will be measured at weeks 0, 12, and 24. The 36-item Short-Form (SF-36), Chronic Liver Disease Questionnaire-NAFLD (CLDQ-NAFLD), and Traditional Chinese Medicine Syndrome Score Scale (TCMSSS) will be administered at weeks 0 and 24. The TCMSSS consists of seven questions, each divided into four grades according to their frequency or degree of occurrence. TCM syndrome diagnosis and its degree of correlation can be reflected by the combination and degree of positive symptoms.

Medication will be issued at the end of the follow-up visit. Patients will receive health education and medication for the next 6 weeks, with all remaining medication from the previous visit. The quantity of the remaining drug will be recorded in the CRF. Treatment compliance will be assessed by medication at each visit, and serious adverse events will be recorded. After 24w of treatment, drug distribution will be stopped, and all subjects included in the project will be followed up for 6w. The research progress is illustrated in Table 2.

**Table 2:** Measurement items and points of data capture

Research stage	Enrolment	Allocation	Intervention				Follow up
**Visit Number**	1st	1st	2nd	3rd	4th	5th	6th
**Time point (week)**	-2-0	0	6	12 ± 5d	18 ± 5d	24 ± 5d	30 ± 5d
**Baseline basic information collected**							
Eligibility screen	×						
Informed consent	×						
Demographic information	×						
Medical and treatment history		×					
Combined disease	×	×	×	×	×	×	×
Combined medication	×	×	×	×	×	×	×
Record of diet and exercise		×	×	×	×	×	×
**Intervention**							
DGSY group							
Placebo group							
**Observation indices**							
** Vital sign and anthropometrics**	×	×	×	×	×	×	×
** Imaging**							
MRI-PDFF	×						
CAP, LSM	×	×		×		×	×
** Liver function**							
ALT, AST, GGT, DBIL, TBIL, IBIL	×	×	×	×		×	×
** Blood lipid**							
TC, TG, LDL-C, HDL-C		×		×		×	
** Blood gluose**							
FBG		×		×		×	
FINS		×		×		×	
HOMA - IR		×		×		×	
** Life quality questionnaire**							
SF-36		×				×	×
CLDQ-NAFLD		×				×	×
TCMSSS		×				×	×
**Safety observations**							
Urine pregnancy test	×						
Blood routine		×		×		×	
Urine routine		×		×		×	
Renal function		×		×		×	
Adverse event			×	×	×	×	×

*DGSY* Danggui Shaoyao Powder, *MRI-PDFF* magnetic resonance imaging-proton density fat fraction, *CAP* controlled attenuation parameter, *LSM* liver stiffness measurement, *ALT* alanine aminotransferase, *AST* aspartate aminotransferase, *GGT* gamma-glutamyl transpeptidase, *DBIL* direct bilirubin, *TBIL* total bilirubin, *IBIL* indirect bilirubin, *TC* total cholesterol, *TG* triglyceride, *LDL-C* low-density lipoprotein cholesterol, *HDL-C* high-density lipoprotein cholesterol, *FBG* Fasting blood glucose, *FINS* fasting insulin, HOMA-IR homeostatic model assessment for insulin resistance index, *SF-36* 36-item Short-Form, *CLDQ-NAFLD* Chronic Liver Disease Questionnaire - NAFLD, *TCMSSS* Traditional Chinese Medicine Syndrome Score Scale

### Outcome measures

All the clinical indicators observed in this study are as follows: (1) general information: diet and exercise records, age, sex, height, weight, blood pressure, past medical history, and medication history; (2) quality of life assessment: SF-36, CLDQ-NAFLD, and TCMSSS; and (3) laboratory indices: blood routine, urine routine, liver function, kidney function, blood lipid, blood glucose, and insulin resistance index. (4) Imaging indexes: proton density fat fraction MRI-PDFF, CAP, and liver stiffness measurement (LSM) measured by FibroScan.

#### Primary outcome

The primary outcome of this study is the relative change in MRI-PDFF score from baseline to 24 weeks.

#### Secondary outcomes

Secondary outcomes included (1) serum alanine aminotransferase (ALT) activity; (2) blood lipid: triglyceride (TG), total cholesterol (TC), low-density lipoprotein cholesterol (LDL-C), and high-density lipoprotein cholesterol (HDL-C); (3) blood glucose: fasting blood glucose (FBG), fasting insulin (FINS), and homeostatic model assessment for insulin resistance index (HOMA-IR); (4) LSM value measured by FibroScan; and (5) anthropometric parameters, including body weight, body mass index, and waist circumference.

#### Safety outcomes and adverse events

Safety indicators include kidney function, blood routine, urine routine, and electrocardiogram.

Adverse events will be recorded in the CRF table during each visit, and subsequent interventions will be performed.

### Data collection, management, and quality control

Data will be managed using the Clinical Research Integration Platform (CRIP) system. Two data managers will enter and proofread the manuscript. To the extent permitted by law, all research findings (including personal data, laboratory testing documents, and CRF) will be confidential. The subject’s name will not appear on the CRF table; only the subject’s initial name and the number assigned at the time of study participation.

### Statistical analysis

SPSS 26.0 statistical software package is used for the statistical processing of the data. If the data conforms to a normal distribution, a t-test will be adopted; otherwise, a non-parametric test will be adopted. The primary outcome will be analyzed for the relative percentage change in MRI-PDFF at the end of the trial using analysis of covariance (ANCOVA) regression analysis and Fisher’s exact test. Sensitivity analysis was conducted using a mixed-effect repeated measure model. Secondary outcomes will be counted using analysis of covariance (ANOVA). The total shedding rate and shedding rate due to adverse events were compared using the chi-square test. Base value equilibrium analysis: Demographic data and other base value indicators were compared using ANOVA or the chi-square test to measure the equilibrium between the treatment and control groups. The p-value set at 0.05 will indicate statistical significance.

Safety analysis: The chi-square test will be used to compare the incidence of adverse events between the two groups and to list the adverse events in this study, normal/abnormal changes in laboratory test results before and after the test, and the relationship between abnormal changes and the test drug.

## Discussion

Clinical practice and basic studies have demonstrated that DGSY might be effective in reducing liver fat, serum transaminase, and blood lipid in NAFLD patients and animals [9, 13, 14]; however, there is still a lack of high-level clinical evidence. Given the positive clinical effects observed by our team and other research groups, it is particularly important to conduct further standardized RCT studies to evaluate its clinical efficacy.

The selection of the main efficacy evaluation indices is one of the key factors for the success of drug clinical trials. Due to the infeasibility of observing clinical outcomes (incidence, survival rate, and all-cause mortality of liver transplantation for cirrhosis), the evaluation of liver histology is generally used as a substitute endpoint for NAFLD clinical trials [15]. As the “gold standard” for histological evaluation of NAFLD, liver biopsy can accurately diagnose the disease and determine the stage of the disease to predict the prognosis of the disease. However, due to the invasive nature of the examination, it may have postoperative risks such as abdominal pain, infection, and high cost, which is generally difficult for NAFLD patients to accept [16]. MRI-PDFF is a biomarker measured by MRI technology for the quantitative assessment of fat in the entire liver with more accurate performance than CAP in the noninvasive diagnosis of hepatic steatosis in NAFLD patients. Many studies have confirmed a good correlation between PDFF values and liver histology [17–19], which has been recognized in recent years as a reliable noninvasive evaluation index for early clinical trials to evaluate the efficacy of NAFLD. Therefore, this study will use the relative changes in MRI-PDFF as the primary outcome. To comprehensively evaluate the efficacy of DGSY, we selected the absolute changes in serum ALT activity, LSM, BMI, blood lipids, blood glucose, and insulin resistance index as the secondary efficacy evaluation index. Relevant studies have shown that the quality of life of patients with NAFLD decreases to varying degrees. Therefore, we will use the SF-36 and CLDQ-NAFLD scales to evaluate the improvement in their quality of life and comprehensively evaluate the clinical efficacy of DGSY in treating NAFLD.

In summary, for the treatment of NAFLD, this study used the classic TCM prescription “Danggui Shaoyao Powder” as the treatment drug and the recognized non-innovative imaging index MRI-PDFF as the main efficacy evaluation index. Through a prospective design, a single-center, randomized, double-blind, placebo-controlled clinical trial was systematically carried out to evaluate the clinical efficacy of this prescription in the treatment of NAFLD.

## Trial status

Recruitment started in February 2020, and it is expected to finish in December 2023.

## Supplementary Information

**Additional file 1.** SPIRIT Checklist for Trials.

## Data Availability

Not applicable.

## Abbreviations

DGSY: Danggui Shaoyao Powder
MRI-PDFF: Magnetic resonance imaging-proton density fat fraction
CAP: Controlled attenuation parameter
LSM: Liver stiffness measurement
ALT: Alanine aminotransferase
AST: Aspartate aminotransferase
GGT: Gamma-glutamyl transpeptidase
DBIL: Direct bilirubin
TBIL: Total bilirubin
IBIL: Indirect bilirubin
TC: Total cholesterol
TG: Triglyceride
LDL – C: Low density lipoprotein
HDL – C: High density lipoprotein
FBG: Fasting blood glucose
FINS: Fasting insulin
HOMA – IR: Homeostatic model assessment for insulin resistance index
SF-36: 36-Item Short-Form
CLDQ – NAFLD: Chronic Liver Disease Questionnaire – NAFLD
TCMSSS: Traditional Chinese Medicine Syndrome Score Scale
CRF: Case Report Form
